# Characterization of a Novel Regulator of Biofilm Formation in the Pathogen *Legionella pneumophila*

**DOI:** 10.3390/biom12020225

**Published:** 2022-01-27

**Authors:** Courtney Marin, Ogan K. Kumova, Shira Ninio

**Affiliations:** 1 Department of Microbiology and Immunology, Drexel University College of Medicine, Philadelphia, PA 19129, USA; courtmarin13@gmail.com (C.M.); Ogan.kumova@fda.hhs.gov (O.K.K.); 2 Kinneret Limnological Laboratory (KLL), Israel Oceanographic and Limnological Research (IOLR), Migdal 14950, Israel

**Keywords:** *Legionella pneumophila*, biofilm, motility, growth rate, cellular replication, virulence

## Abstract

*Legionella pneumophila* is a Gram-negative, facultative intracellular pathogen that causes severe pneumonia known as Legionnaires’ disease. The bacterium causes disease when contaminated water is aerosolized and subsequently inhaled by individuals, which allows the bacteria to gain access to the lungs, where they infect alveolar macrophages. *L. pneumophila* is ubiquitous in the environment, where it survives by growing in biofilms, intracellularly within protozoa, and planktonically. Biofilms are a major concern for public health because they provide a protective niche that allows for the continuous leaching of bacteria into the water supply. In addition, biofilms enhance the survival of the bacteria by increasing resistance to temperature fluctuations and antimicrobial agents. Currently, there is little known about biofilm formation and regulation by *L. pneumophila*. Here, we present evidence of a specific gene, *bffA*, which appears to be involved in the regulation of motility, biofilm formation, cellular replication, and virulence of *L. pneumophila*. A strain lacking *bffA* has an enhanced biofilm formation phenotype, forming biofilms that are both faster and thicker than wild type. Additionally, the knockout strain has significantly reduced motility, enhanced uptake into amoebae, and altered growth kinetics on solid media. Our data suggest a potential role for *bffA* in signaling pathways that govern changes in growth rate and motility in response to environmental conditions.

## 1. Introduction

*Legionella pneumophila* is an opportunistic pathogen and the causative agent of a severe form of pneumonia known as Legionnaires’ Disease. Legionnaires’ Disease is responsible for 18,000 hospitalizations per year in the United States, has a case mortality rate of 5–30%, and is particularly problematic for elderly and immunocompromised individuals [1,2,3,4,5,6]. The disease is caused when a contaminated water source becomes aerosolized and is inhaled by humans, thereby providing bacteria access to the lungs, where they infect alveolar macrophages [2,7,8]. *L. pneumophila* is ubiquitous in aquatic environments, where it can be found planktonically, intracellularly within protozoa, or within biofilms [9]. 

In recent years, biofilms have become a major public health concern with the increased awareness of the potential of biofilms to provide a continuous source of bacterial contamination in freshwater supply systems. Importantly, association with biofilms has been shown to enhance the survival of residing bacteria [4,10,11] by providing protection against antibiotics, antimicrobial agents, temperature fluctuations, and nutrient and oxygen limitations [1,4,12,13,14,15]. The presence of biofilms containing *L. pneumophila* in domestic water supplies has been identified as a potential cause of drinking water-acquired Legionellosis [10]. *L. pneumophila* is responsible for up to 30% of drinking water-borne diseases each year, and up to 80% of drinking water deaths in the United States [16]. Additionally, a large percentage of nosocomial-acquired Legionellosis is due to inadequate sanitization of water supply systems and the inability to eliminate biofilms within the system [5]. 

The identification of key regulators could prove useful in the development of novel antibiofilm strategies that would reduce the incidence of disease by eradicating biofilms from water supplies. While there has been some research into *Legionella* mono-and multispecies biofilms [6,9,10,12,15,17,18,19,20,21,22,23,24], the majority of the genes and proteins involved in the biofilm pathway of *L. pneumophila* remain to be identified. Of special interest is the second messenger cyclic diguanosine monophosphate (c-di-GMP), which has been shown in other bacteria to play a role in regulating the biofilm lifestyle [25,26,27]. A few of the *L. pneumophila* genes involved in the metabolism of c-di-GMP have been identified [18,19,28,29]. In a paper by Levi et al., the authors presented data showing that deletion of putative diguanylate cyclase (DGC) and phosphodiesterase (PDE) genes did not have any effect on intracellular growth; however, the overexpression of some of the genes led to an intracellular growth defect. This work provides evidence that c-di-GMP levels affect the virulence of *L. pneumophila*. Carlson et al. show that a heme nitric oxide (H-NOX) regulates the activity of a DGC, lpg1057. Upon deletion of the *hnox1* gene, the bacteria display a hyper-biofilm phenotype, suggesting that *hnox1* is inhibiting the activity of lpg1057 to block biofilm formation. Thus, when *hnox1* is deleted, intracellular levels of c-di-GMP increase, leading to the hyper-biofilm phenotype observed. These findings demonstrate the importance of c-di-GMP pathways in *L. pneumophila*, linking the pathway to biofilm regulation and intracellular survival of this pathogen.

In this work, we sought to investigate the function of a previously uncharacterized *Legionella* gene *lpg1387* containing a putative phosphodiesterase (PDE) domain. We found that a deletion mutant of *lpg1387* has altered growth kinetics on solid media, enhanced uptake into amoebae, decreased flagellar motility, and enhanced biofilm formation compared to wild type *L. pneumophila*. Our data suggest that the gene, which we named *bffA*, is involved in the regulation of motility and biofilm formation in *L. pneumophila*.

## 2. Materials and Methods

### 2.1. Strains, Plasmids, and Primers

The strains used in this study are outlined in Table 1. We used both *L. pneumophila* strains, CR39 and JR32, and generated a Δ*bffA* mutant in both backgrounds. In-frame deletion of *bffA* was performed by homologous recombination after the introduction of the suicide vector pSR47s (pCM4) containing a fusion of the 800 bp upstream and 800 bp downstream of *bffA* generated using primers SN252, SN253, SN254 and SN255 and ligated with plasmid pSR47s digested with XbaI and SacI. Plasmid pMMB207-Cam with full-length *bffA* (pCM2) was generated by digesting the plasmid pCM1 with BamHI and HindIII and ligating with a PCR fragment generated using primer SN256 and SN257. The plasmid pMMB207-empty (pCM1) was generated by digesting pMMB207-M45NT with BamHI and HindIII and treating with T4 DNA ligase to fill the HindIII site, followed by blunt-end ligation and transformation. All vectors were confirmed by PCR and digest. See Table 1 for full list of strains, primers, and plasmids.

### 2.2. Media and Antibiotics

For *Legionella* strains: all were grown in either AYE broth [1% yeast extract [HIMEDIA], 1% *N*-(2-acetamido)-2-aminoethanesulphonic acid (ACES; pH 6.9) [Amresco], 3.3 mM L-cysteine [Sigma], 0.33 mM Fe(NO3)_3_] or on charcoal yeast extract agar (CYE) (AYE containing 1.5% bacto-agar [HIMEDIA], 0.2% activated charcoal [Sigma]). Antibiotics, all purchased from Sigma unless stated otherwise, used for *Legionella* strains were added to the media at the following concentrations: streptomycin 100 μg/mL, kanamycin 20 μg/mL, chloramphenicol 10 μg/mL.

*E. coli* strains were grown in LB medium (LB) 1% bacto-tryptone, 0.5% yeast extract, 1% NaCl [Sigma] or on LB-agar plates (LB containing 1.5% bacto-agar). Antibiotics were added to the media at the following concentrations: ampicillin 100 μg/mL, kanamycin 40 μg/mL, chloramphenicol 25 μg/mL.

*Acanthamoeba castellanii* (ATCC 30234) were grown at room temperature in ATCC medium 712 (PYG). One hour before infection, *A. castellanii* cultures were incubated at 37 °C in a 5% CO_2_ atmosphere in PYG medium without glucose, protease peptone, and yeast extract (amoebae buffer). If indicated, after infection, *A. castellanii* was washed with sterile phosphate-buffered saline (PBS).

### 2.3. Broth Growth Curve

Broth growth curves were started from plate-grown bacteria that had been incubated for 48 h. Overnight cultures grown in liquid AYE medium were diluted to OD_600_ = 0.05 and grown shaking at 37 °C in glass culture tubes, with OD_600_ measurements taken approximately every 45 min. 

### 2.4. Intracellular Growth Curve in A. Castellanii 

*A. castellanii* was plated at a concentration of approximately 1 × 10^5^ cells per well of a 24-well plate, 24 h prior to infection. One hour before infection, each well was washed twice with sterile PBS and the media was replaced with amoebae buffer, and the plates were incubated at 37 °C. Plate-grown bacteria that had been incubated for 48 h prior to infection were suspended in distilled water to OD_600_ = 1.0 and then diluted to OD_600_ = 0.05. Each well was infected with 5.0 μL of the OD_600_ = 0.05 suspension, a multiplicity of infection (MOI) of 1, and incubated for two hours at 37 °C. Time-zero wells were processed by removing the media, washing the wells three times with sterile PBS to remove extracellular bacteria, and lysing the amoebae in 1.0 mL distilled water, then diluting and plating for enumeration of colony-forming units (CFU). The rest of the wells were washed three times with sterile PBS and 1.0 mL fresh media was added to each well. The plates were returned to the incubator and processed at the specified times. To process the subsequent time points, the media from each well was combined with the lysate to account for extracellular, as well as intracellular, bacteria.

### 2.5. Flagellar Motility Assay

Overnight cultures of each strain were serial diluted 6:7 from an initial OD_600_ of 0.5, (37 °C), or 0.65 (30 °C), and grown shaking at 37 °C or 30 °C. The OD_600_ of each culture was read in the morning, and the motility was observed using a Nikon Ti Eclipse microscope. Each culture was given a motility score based upon the percentage of the observable culture performing directional motility. To calculate the motility score, each culture was observed for several minutes over five fields of view per sample, to enumerate a percentage of the overall culture that was either stationary or moving in a directional manner. Bacteria were manually counted, with an average of 50–100 bacteria per field of view to generate the percentage of motile bacteria per culture. The experiment was repeated three independent times and statistical analysis was performed using an unpaired t-test.

### 2.6. 96-Well Biofilm Assay

Plate-grown strains were resuspended to OD_600_ = 0.3 in AYE. 200 μL of each strain was plated per well of the 96-well plate and then incubated at 30 °C. The wells were stained with 200 μL of 0.1% crystal violet on the indicated days and resolubilized in 30% acetic acid, and absorbance was measured in OD_600_.

### 2.7. Coverslip Biofilm Assay

Cultures of each strain were grown overnight shaking at 37 °C. The OD_600_ in the morning was adjusted to OD = 1.0, and 250 μL was applied to coverslips placed inside each well of a 24-well plate. The plate was then incubated at 30 °C on a slant to allow the air interface to run across the center of the coverslip, according to the method by Merritt et al. [35]. The coverslips were then stained with 4,6-diamidino-2-phenylindole (DAPI), mounted using Mowiol, and imaged on a Nikon T*i* Eclipse microscope. 

### 2.8. Biofilm Binding Assay

To test for enhanced binding of Δ*bffA* to the wells of a 96-well plate, we grew wild-type and Δ*bffA* strains overnight shaking at 37 °C. In the morning, the cultures were diluted to OD_600_ = 1.0 and 200 μL was plated per well in 9 wells for each strain. For T0, the bacteria were allowed to adhere for 15 min, 2 h, or 4 h before the media was removed and the wells were washed with distilled water. The adherent bacteria were collected from the well using a sterile cotton-tipped applicator, resuspended in sterile PBS, and plated for CFUs. 

### 2.9. Gentamicin Protection Assay

To measure uptake into *A. castellanii*, amoebae were plated at a concentration of 1 × 10^5^ cells per well of a 24-well plate one day prior to infection. One hour prior to infection, the wells were washed with PBS and the amoebae were incubated with amoebae buffer at 37 °C for one hour. The wells were then infected with an MOI = 100 for 2 h, following a 5 min centrifugation at 1000 rpm. After 2 h, the amoebae were washed with PBS containing 100 μg/mL gentamicin and subsequently incubated for 30 min at 37 °C with buffer containing 100 μg/mL gentamicin. The lysate was collected and plated for CFUs. Additionally, the input was plated for CFUs to calculate the percentage uptake of each strain. Statistical analysis was performed using an unpaired t-test.

## 3. Results

### 3.1. L. pneumophila ΔbffA Exhibits Altered Growth Kinetics on Solid Medium, but Not in Broth, Compared to Wild-Type

**Figure 1:**
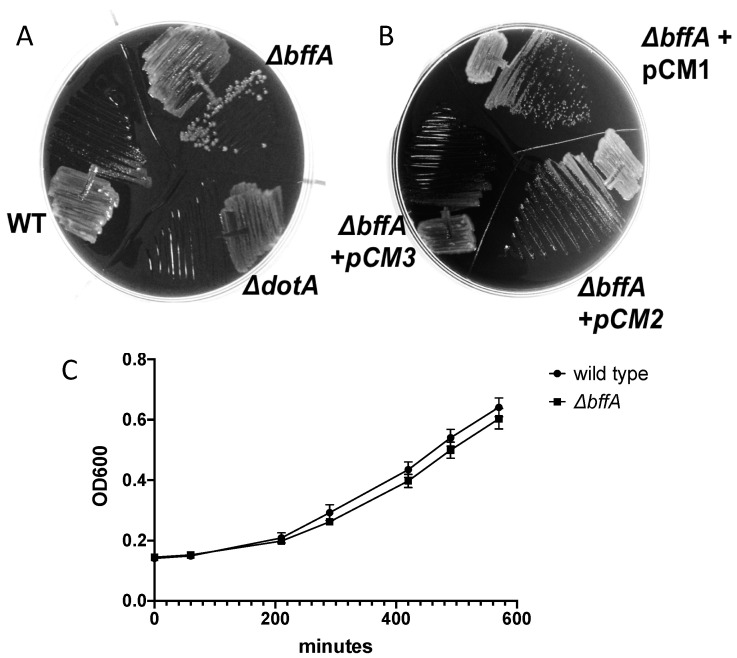
ΔbffA forms single colonies in two days compared to wild-type and ΔbffA +pBffA (pCM2) and shows no difference in planktonic growth compared to wild-type. (**A**) Wild-type, ΔbffA, and ΔdotA strains after two days of growth at 37 °C on CYE agar. (**B**) ΔbffA was also grown with the addition of empty vector (pCM1), pBffA (pCM2), or pBffA_(1-366)_ (pCM3). Both plates were incubated in the same incubator simultaneously, and all strains are in the CR39 genetic background. The image is of a representative experiment. (**C**) Broth growth curves performed with wild-type and ΔbffA strains of the CR39 background display no difference in growth kinetics by the ΔbffA strain. 10 h growth curve. The graph is an average of six replicates.

*bffA* is annotated as a putative phosphodiesterase, however, it appears to be lacking key residues that would be involved in the cleavage of c-di-GMP (Figure S1), suggesting it lacks the ability to cleave c-di-GMP but may still be involved in a signaling pathway that responds to fluctuations in intracellular levels of the second messenger. Phosphodiesterases (PDEs) cleave c-di-GMP to produce 5’-phosphoguanylyl-(3’,5’)-guanosine (pGpG), which is then hydrolyzed to produce two GMP molecules. PDEs are known to be involved in lifestyle decisions in many bacterial species, including *Pseudomonas aeruginosa* and *Vibrio cholerae*, where c-di-GMP signaling regulates both planktonic and biofilm growth [13,26,27,36,37,38,39]. While we have no evidence that *bffA* is acting as a PDE, the first observation we made with the Δ*bffA* strain, once it was constructed, was that it forms single colonies in 48 h, compared to 72–96 h required for wild-type bacteria to form single colonies (Figure 1A). This phenotype is also observed when the Δ*bffA* strain is grown from a frozen stock, where it takes an additional 24 h for the wild-type strain to grow. Furthermore, we were able to revert the Δ*bffA* strain to a wild-type phenotype using a pMMB207 derived expression vector, where the expression of *bffA* is driven off of the *icmR* promoter (Figure 1B) when the proper selection was present. The 3′ end of the *bffA* gene, encoding C-terminal residues 367–469 of the BffA protein, is not necessary for the observed growth regulation activity of *bffA*, as a vector encoding for a BffA truncation is sufficient to revert the mutant phenotype back to wild-type growth rates (Figure 1B). The complementation observed is specifically due to the expression of *bffA*, and not merely the presence of the plasmid since the empty vector pCM1 did not complement the growth phenotype. These results suggest that *bffA* has a role in the regulation of *L. pneumophila* growth. Additionally, these results indicate that the C-terminus of BffA is not required for the observed growth phenotype.

The Δ*bffA* mutant was also characterized in liquid broth to test whether the growth rate phenotype we observed was specific to solid media. The Δ*bffA* strain did not display altered growth kinetics in broth when compared to wild-type (Figure 1C). This suggests that *bffA* is important to growth on solid surfaces, but not during the planktonic growth phase of the bacteria.

### 3.2. The ΔbffA Strain Has Normal Rates of Intracellular Growth in A. Castellanii 

**Figure 2:**
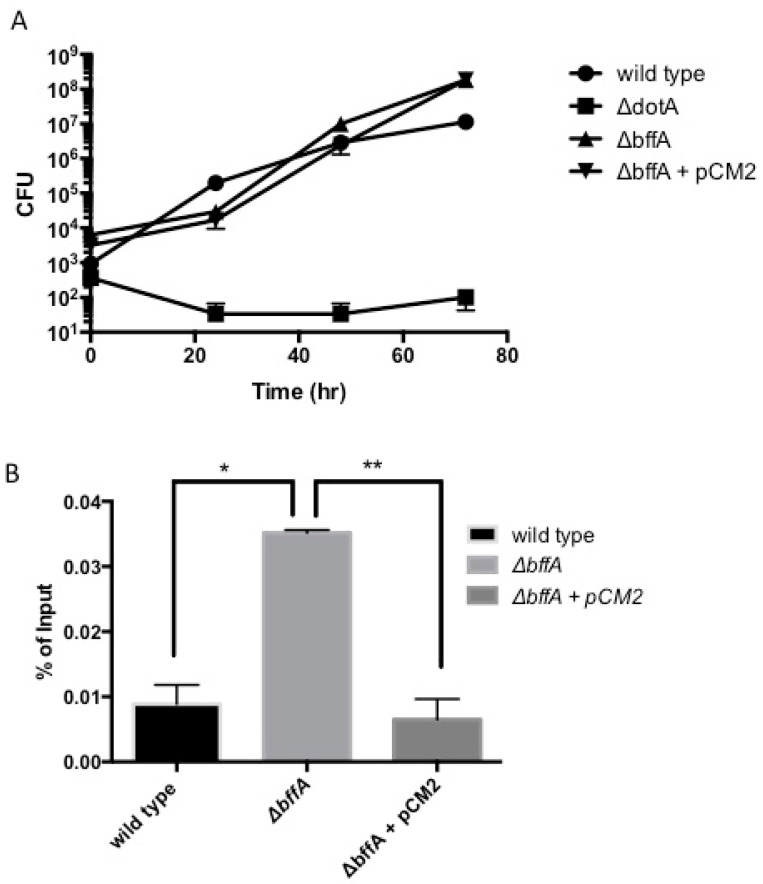
Δ*bffA has normal intracellular growth but enhanced uptake into amoebae*. Intracellular growth in amoebae was tested using a 72-hour amoebae growth curve (**A**). To test the enhanced uptake, we performed a gentamicin protection assay (**B**). * p < 0.05, ** p < 0.005 Experiments are an average of three repetitions.

*L. pneumophila* is an intracellular pathogen, with a large portion of its lifecycle spent replicating within a *Legionella*-containing vacuole inside of protozoans in the environment, or macrophages during human infection [2,40,41,42,43,44,45,46,47,48]. Since we observed altered growth kinetics on solid media, we determined the intracellular growth rate of Δ*bffA* in *A. castellanii*, which is considered a natural host for *L. pneumophila* in the environment. We found that in *A. castellanii*, Δ*bffA* has a slight enhancement in intracellular growth rate compared to wild-type (Figure 2A). We were unable to see the reversal of the Δ*bffA* phenotype to a wild-type phenotype in this assay due to the inability to maintain adequate levels of antibiotics in the media over the three-day time course. The complementation strain rapidly loses the vector carrying *bffA* within 48 h of inadequate antibiotic levels, making complementation impossible. We have observed that, when grown on CYE without antibiotics, the complemented strain forms single colonies in two days, and these colonies no longer possess the ability to grow on antibiotic-containing plates, indicating they have lost the complementation vector (not shown). Additionally, through these experiments, we observed a large increase in the uptake of the Δ*bffA* strain into the amoebae at time zero, which is reverted to wild-type levels with the expression of *bffA in trans*. We further tested uptake levels using a gentamicin protection assay, in which bacteria that are not taken up by *A. castellanii* are killed by the addition of the antibiotic gentamicin to the amoebae medium. For this assay, amoebae are infected with a high multiplicity of infection (MOI), and the output, i.e., what is collected from the lysed amoebae after antibiotic treatment, is normalized to the input to generate a percentage of the input that is protected from antibiotic treatment. Figure 2B shows that Δ*bffA* has significantly enhanced uptake into *A. castellanii* and the addition of *bffA in trans* reverted the uptake rate back to wild-type levels, indicating a phenotype specific to the deletion of *bffA*. The increased uptake phenotype of Δ*bffA* could be a manifestation of the altered growth rate during the short infection. Alternatively, *bffA* may function in the downregulation of uptake into amoebae through a yet-unknown mechanism. 

### 3.3. ΔbffA Forms Biofilms Faster than the Wild-Type Strain

**Figure 3:**
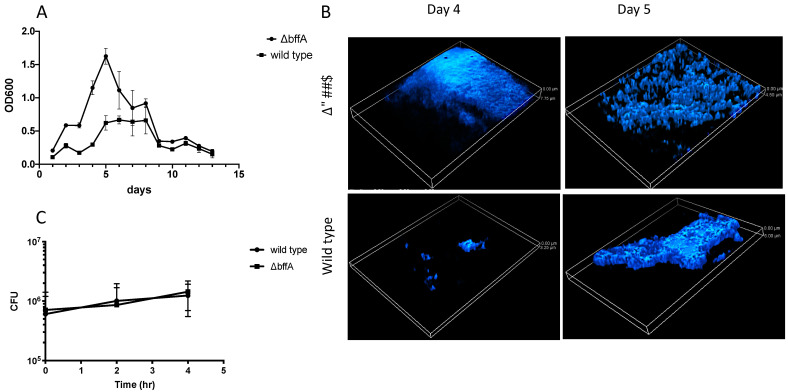
Δ*bffA forms biofilms faster than wild type*. Biofilms grown in 96-well plates (**A**) and on glass coverslips and stained with DAPI (**B**). Binding assay (**C**) to test the ability of the bacteria to adhere to the 96-well plate. All experiments were repeated 3 times, with A and B a representative of each, and C the average of the three repetitions.

In many bacterial species, including *P. aeruginosa* and *V. cholerae*, planktonic growth and biofilm growth are regulated in an opposing manner [49,50,51]. Based upon this and on our previous experiments with the Δ*bffA* strain, we hypothesized that the deletion of *bffA* would lead to a shift in the growth of the Δ*bffA* strain to a biofilm lifestyle, as opposed to a motile, planktonic lifestyle. Since we have already observed alterations in growth on solid media and virulence, we sought to establish several biofilm assays to test the ability of Δ*bffA* to form biofilms. In a 96-well biofilm formation assay, plate grown bacteria were resuspended to an OD_600_ = 0.3 and plated at 200 μL per well of a 96-well plate. For each time point during the 12 days of the experiment, washed wells were stained with 0.5% crystal violet to measure the degree of biofilm formation. The crystal violet was then resolubilized from the stained biofilms in 30% acetic acid, and the OD_600_ was measured. We observed that all biofilms formed at the air-liquid interface, and the Δ*bffA* strain formed biofilms more rapidly and densely than the wild-type strain (Figure 3A). This indicates that *bffA* may be involved in the regulation of biofilm formation. Attempts to complement this phenotype were unsuccessful due to the instability of the complementation vector in the Δ*bffA* strain.

We next established an assay for the qualitative visualization of the biofilms. For this assay, we grew the biofilms on glass coverslips in a 24-well plate, tilted to ensure the air interface of the culture was situated across the center of the coverslip. The coverslips were removed from the plate and stained to observe the structure of the biofilm and measure biofilm thickness by fluorescence microscopy. We observed that Δ*bffA* biofilms formed earlier and appeared thicker than wild-type biofilms (Figure 3B). These results are in line with the results obtained using the 96-well plate assay.

To test whether the enhanced biofilm formation may be a result of differences in the capacity of the strains to bind to the wells of the 96-well plate, we performed a plate-binding assay. Bacteria were plated at 200 μL of OD_600_ = 1.0 per well of a 96-well plate and allowed to adhere to the wells for 15 min, 2 h, and 4 h. We did not observe any difference in the ability of Δ*bffA* to bind to the wells compared to the wild-type strain (Figure 3C). We concluded that the Δ*bffA* strain is able to form biofilms faster than the wild-type, in a mechanism that is independent of the initial adherence phase of biofilm formation.

### 3.4. ΔbffA Is Less Motile than Wild-Type Bacteria at 37 °C, but Not at 30 °C

**Figure 4:**
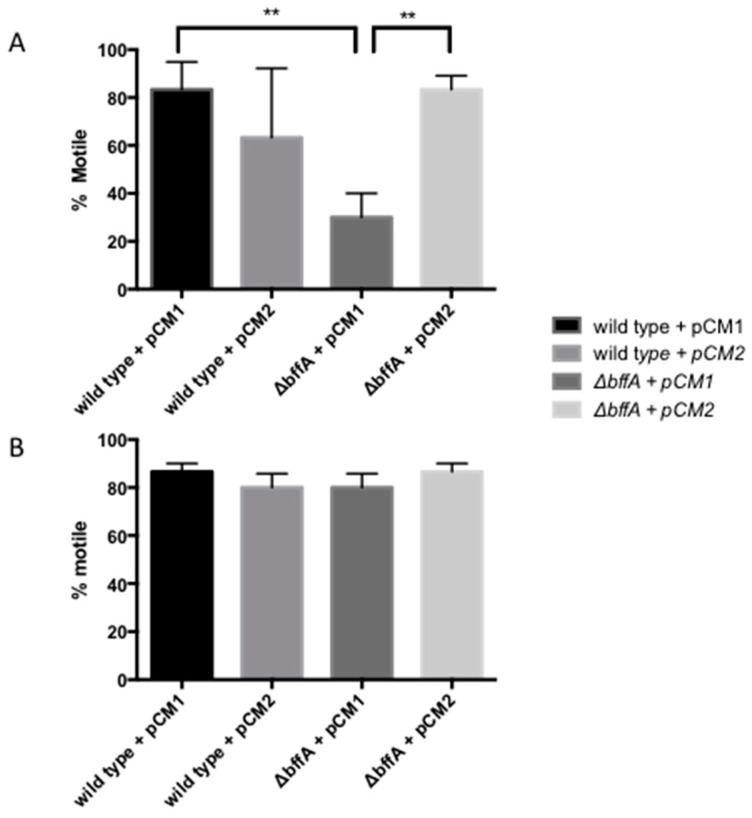
Δ*bffA has reduced flagellar motility at 37 °C, but not 30 °C*. Flagellar motility assays performed at 37 °C with the JR32 strains (**A**) and 30 °C (**B**) as detailed in the materials and methods. ** *p* < 0.005, *N* = 3.

Motility and biofilm formation are inversely regulated in bacteria [26,27,49,52], so we sought to determine if we could observe reduced motility in the Δ*bffA* strain since we previously observed enhanced biofilm formation compared to wild-type. With the lack of a plate-derived motility assay, we used light microscopy to measure the percentage of the population displaying flagellar motility in several overnight cultures. Using this approach, we determined the level of motility in the wild-type and Δ*bffA* strains, as well as in wild-type and Δ*bffA* harboring pCM2. We set up 12 cultures and 6:7 serial dilutions of each strain, starting with an OD_600_ = 0.5. By setting up a series of cultures, we expected that we would be able to capture the bacteria at peak motility. The cultures were grown overnight at 37 °C, and the OD_600 was_ measured again in the morning, followed by subsequent microscopic analysis for motility. We observed that wild-type, wild-type-pCM2, and Δ*bffA*–pCM2 were able to reach peak motility levels of 90% (Figure 4A), independent of culture density. On the other hand, the Δ*bffA* strain had very limited motility, regardless of culture density, and the highest level of motility we were able to record for this strain was 20% of the culture displaying flagellar motility (Figure 4A). 

Interestingly, when repeating this assay with bacteria grown at 30 °C, we observed no difference in flagellar motility between the Δ*bffA* strain and the other strains (Figure 4B). All of the strains tested showed 80–90% motility of the culture, with no significant difference in motility detected between the strains (*p* < 0.005). This suggests a temperature-dependent role for *bffA* in the regulation of flagellar motility.

## 4. Discussion

In recent years, the bacterial second messenger, c-di-GMP, has become the focus of much research and found to influence biofilm formation, motility, cellular replication, and virulence in many bacterial species. Work aimed at identifying proteins involved in the metabolism of c-di-GMP has led to the discovery of degenerate DGCs and PDEs that still bind c-di-GMP, though they lack enzymatic activity [38,50,53,54]. In these studies, proteins were found to be missing specific motifs and/or residues required for the production of c-di-GMP or its breakdown. While degenerate c-di-GMP metabolizing genes would appear to be non-functional, their deletion mutants are often found to have strong phenotypes [38,53,54]. Here, we investigate an *L. pneumophila* protein we termed BffA, which contains a putative PDE domain but lacks conserved residues found in known proteins from this family. In our work, we show that the deletion of *bffA* results in strong phenotypes linked to the regulation of motility, virulence, cellular replication, and biofilm formation. Our work suggests that BffA may be functioning within the c-di-GMP pathway, despite lacking key PDE active-site residues. 

It has been previously demonstrated that degenerate c-di-GMP metabolism genes can still affect c-di-GMP levels without being enzymatically active. Studies with PelD and FimX from *P. aeruginosa* [38,54] and LapD from *Pseudomonas fluorescens* [53] provide evidence that proteins possessing degenerate DGC and PDE domains maintain the ability to bind c-di-GMP and affect downstream signaling of the molecule. In the case of PelD, the protein possesses a degenerate DGC domain, but still maintains the ability to bind c-di-GMP at the I-site [54]. FimX has a degenerate DGC and PDE domain and can bind c-di-GMP through its PDE domain. Deletion of *fimX* leads to a complete abrogation of biofilm formation, suggesting a major role for c-di-GMP binding proteins in the regulation of motility and biofilm formation [38]. LapD from *P. fluorescens* is also a dual DGC–PDE domain protein, with both domains degenerate and enzymatically inactive. LapD binds c-di-GMP through its degenerate PDE domain and induces biofilm formation through the production and localization of LapA, an adhesin that is essential for the attachment of the bacteria to surfaces [53].

In a recent study, it was reported that an ATPase gene (*mshE*) in *V. cholerae* and its homolog in *P. aeruginosa* lacked all conserved motifs of PDEs, but still functioned as c-di-GMP binding proteins. Upon deletion of *mshE* from *V. cholerae*, the strain had reduced motility and enhanced biofilm formation, much like Δ*bffA*. This suggests that, while it does not appear to have a functional PDE domain, it is possible that BffA is still exerting an effect on c-di-GMP levels within *L. pneumophila*. In a different study with *V. cholerae*, researchers deleted a PDE encoding gene, *cdgJ*, which resulted in reduced motility and enhanced biofilm formation in the knockout strain [49]. However, they were unable to detect any change in global c-di-GMP levels with the deletion of *cdgJ*, suggesting that changes in local levels of the dinucleotide can lead to significant changes within the bacterial cell [49]. A similar finding is reported in a study by Pécastaings et al. [29]—their work characterizes the GGDEF-EAL domain, which contains proteins from the *Legionella* strain Lens—demonstrating that in some cases deletion mutants resulted in hyper biofilm formation without the expected elevation in c-di-GMP levels and suggesting that alternative mechanisms of biofilm regulation exist that do not involve the elevation of global c-di-GMP levels.

The *bffA* deletion mutant displays a phenotype resembling that of the *hnox1* deletion characterized by Carlson et al. [18], both resulting in hyper biofilm phenotypes. The Hnox1 protein was shown to inhibit the diguanylate cyclase activity of Lpg1057 in response to nitric oxide, leading to lower c-di-GMP levels and less biofilm formation [18]. Similarly, BffA may also be responding to a yet-unidentified specific signal within the cell, providing a regulatory pathway-controlling biofilm formation. In fact, a genome-wide transcription analysis carried out by Hochstrasser et al. [28] points at *bffA* as one of a few hundred genes that are regulated by the *L. pneumophila* Lqs–LvbR quorum-sensing regulatory network. Similar to Δ*bffA*, the Δ*LvbR* mutant also results in hyper biofilm formation. Interestingly, in deletion, mutants of both *lvbR* and *lqsR* downregulation of *bffA* are observed in sessile bacteria [28]. This data provides a possible link between quorum sensing and the function of bffA in the downregulation of biofilm formation.

The bffA gene has not been identified in a screen previously performed for the identification of c-di-GMP metabolizing genes in *L. pneumophila* [55]. The screen was designed to identify *L. pneumophila* genes that possess conserved signature sequences linking them to the c-di-GMP pathway and not meant to identify degenerate genes [18,19,55]. To date, only one degenerate PDE gene has been identified in *L. pneumophila*, as part of an operon believed to be orthologous to the *lapD-lapG* system of *P. fluorescens*. The *Legionella* LapD-like protein was studied in vitro, but the effect of the protein has not been demonstrated in vivo [19]. The work presented here is the first example of a degenerate PDE gene in *L. pneumophila*, with a phenotype suggesting a link to the c-di-GMP second messenger signaling pathway. While the mechanism of action of BffA remains to be determined, our data strongly suggest that BffA is a regulatory protein, as we observe inverse regulation of motility and biofilm formation in a *bffA* deletion mutant. Moreover, the expression of *bffA* in trans is able to rescue the motility defect seen in the mutant, indicating that the observed phenotype is specific to the loss of *bffA*, and ruling out the possibility of a genetic polar effect. It remains to be determined whether *bffA* is functioning within the c-di-GMP signaling pathway or through a different regulatory network. Future work will determine the mechanism by which *bffA* regulates lifestyle decisions in the pathogen *L. pneumophila*.

## Figures and Tables

**Table 1:** Strains, Plasmids, and Primers.

SN#	Genotype	Reference	
CR39	*L. pneumophila* serogroup 1, strain LP01 *rpsL*	[30]	
CR58	LP01 *rpsL ΔdotA*	[31]	
JR32	*L. pneumophila* serogroup 1, *rpsL*	[32]	
SN300	LP01 rpsL ΔbffA	This study	
SN306	LP01 *rpsL ΔbffA* +pCM1	This study	
SN307	LP01 *rpsL ΔbffA*+ pCM2	This study	
SN308	LP01 *rpsL ΔbffA* + pCM3	This study	
SN278	JR32 Δ*bffA*	This study	
SN283	JR32 Δ*bffA* + pCM2	This study	
SN284	JR32 Δ*bffA* + pCM3	This study	
SN286	JR32 Δ*bffA* + pCM1	This study	
**Plasmid**	**Important properties**	**Marker**	**Reference**
pMMB207-M45NT	Amino-terminal M45 epitope tag vector	Cm	[33]
pSR47s	Gene replacement vector	Kan	[34]
pCM1	pMMB207 empty vector-lacking PstI site in MCS	Cm	This study
pCM2	pCM1 with full length bffA	Cm	This study
pCM3	pMMB207-M45T with bffA_(1-366)_	Cm	This study
pCM4	pSR47s-bffA	Kan	This study
**Primers**	**Sequence**		**Sites**
SN252	*GCGTCTAGA*ATTTATTGTCCTTATTTTTATAGTC		XbaI
SN253	CGGGTAGGAGCAAATATTTACTTCAATCATAACG		
SN254	*TAAATATTTG*CTCCTACCCGAGGTGCTTAA		
SN255	*CGCGAGCTC*AACCTGCTTTGCTAAAACAAGA		SacI
SN256	*GCGGGATCCttATGATTGAAGTAAATATTTGGTTA*		BamHI
SN257	*gcgaagcttTTAAGCACCTCGGGTAGGA*		HindIII

## Data Availability

Not applicable.

## Supplementary Materials

The following are available online at https://www.mdpi.com/article/10.3390/biom12020225/s1, Figure S1: Schematic diagram showing the location of the putatitive phosphodiesterase (PDE) domain of *bffA*.
