# Supplementary material for: Characterization of a Novel Regulator of Biofilm Formation in the Pathogen Legionella pneumophila
Source: Biomolecules. 2022 Jan 27;12(2):225. doi: 10.3390/biom12020225 (PMC8961574; doi:10.3390/biom12020225)
Supplement: Supplementary file 1 [file biomolecules-12-00225-s001.zip › biomolecules-1561342-supplementary.pdf]

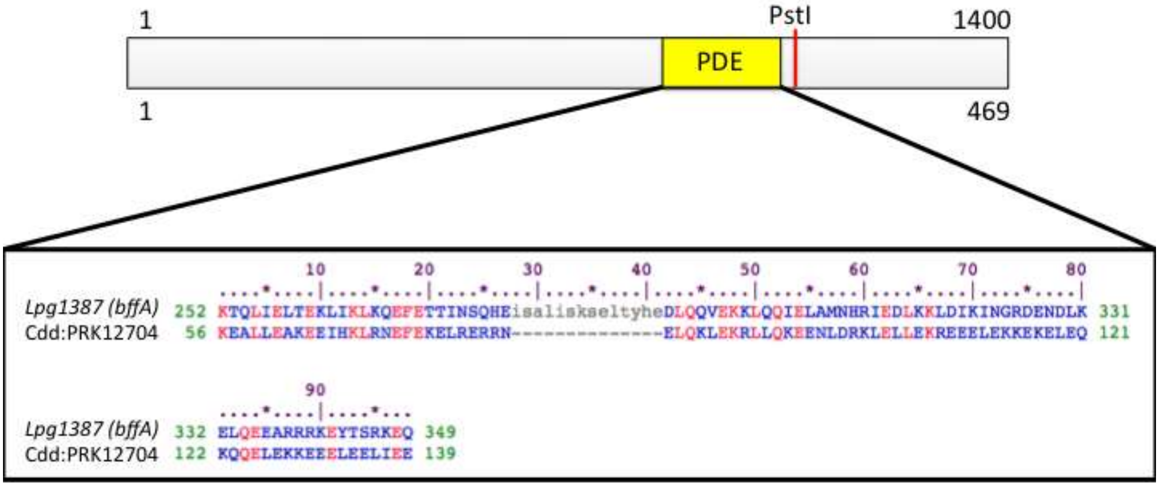

Figure S1. Schematic diagram showing the location of the putatitive phosphodiesterase (PDE) domain of *bffA.* Expanded out, the putative PDE domain sequence is aligned with conserved domain database (CDC) domain PRK12704 from *Haemophilus influenzae* [1].

Reference:

1. Lu, S.; Wang, J.; Chitsaz, F.; Derbyshire, M.K.; Geer, R.C.; Gonzales, N.R.; Gwadz, M.; Hurwitz, D.; Marchler, G.H.; Song, G.S.; et al. *CDD/SPARCLE: the conserved domain database in 2020. Nucleic Acids Res.* **2020**, *48*, D265–D268.
